# Occupational safety and health in the agricultural sector: a bibliographic review

**DOI:** 10.47626/1679-4435-2023-1137

**Published:** 2023-11-24

**Authors:** Darwin Jose Mendoza Galvis, Adriana Lorena Vega Molina

**Affiliations:** 1 Fundación universitaria del Área Andina, Ingeniería Industrial, Valledupar, Cesar, Colombia

**Keywords:** occupational health, agriculture, policy, salud laboral, agricultura, políticas

## Abstract

In the labor context the agricultural sector has been a little neglected in terms of the implementation of policies and standards that allow maintaining low accident rates, and in the last year strategies have been established to improve this situation. The aim of this study was to conduct a bibliometric analysis of the scientific literature on the methodologies used to plan, verify, and maintain occupational safety and health in the agricultural sector. The methodology focused on the search for information in the Scopus and Web of Science databases through a search equation, and then through inclusion and exclusion criteria to evaluate the selected articles. It was found that the country with most studies on occupational safety and health was the United States, and that methodologies such as social participation, videos, and team learning are among the most successful processes. The year of publication with the largest amount of research published was 2021, the survey approach appears in 82% of the articles, the use of technology as a means of dissemination of improvement actions was evidenced, and the most innovative processes included: use of religion, checklists, fear, threats, and work organization processes.

## Abstract (es)

En el contexto laboral el sector agrícola ha sido un poco relegado en cuanto a la implementación de políticas y normas que permitan mantener índices bajos de accidentabilidad, y en el último año se han establecido estrategias para que esto mejore. El objetivo de este estudio era realizar un análisis bibliométrico de la literatura científica sobre las metodologías empleadas para planear, verificar y mantener la seguridad y salud en el trabajo en el sector agrícola. La metodología se centró en la búsqueda de información en la base de datos Scopus y Web of Science a través de una ecuación de búsqueda, para luego a través de criterios de inclusión y exclusión trabajar con los artículos seleccionados. Se evidenció que el país que más ha trabajado con la seguridad y salud en el trabajo es los Estados Unidos, que han establecido metodologías como participación social, videos y dentro de los procesos más acertados está el aprendizaje en equipo. El año de publicación con la mayor cantidad de investigaciones establecidas fue durante el 2021; la encuesta aparece en el 82% de los artículos, y se evidenció el uso de la tecnología como medio de divulgación de acciones de mejoras y dentro de las más innovadoras estarían, el uso de la religión, lista de comprobación, el miedo y la amenazas y los procesos de organización del trabajo.

## INTRODUCTION

One of the essential pillars pursued by government entities is food safety, and no wonder it is one of the sustainable environmental goals, specifically that of number two, zero hunger,^1^ thus bringing attention to the agricultural sector, which is called to formulate actions aimed to reduce hunger. Furthermore, it is essential for economic growth, accounting from 4% to 25% of the gross domestic product (GDP), allowing for improving the income of the poorest; some indicators point out that 65% of working adults earning their living in the agricultural sector.^2^

Furthermore, the food crisis resulting from COVID-19 and the different social and environmental phenomena that had a direct impact on the agricultural sector have caused negative effects, compelling countries to formulate policies and actions aiming to structure and improve this sector in order to increase its levels of productivity and competitiveness. This has determined the development of projects that somehow attract workers, increasing its labor force, since, according to the International Labor Organization,^3^ the agricultural sector employs sector employs more than one third of the global workforce and is the world’s second largest source of employment.

Conversely, due to agricultural technification, numerous machineries are used, and agriculture is characterized by different types of supplies, geographical and climate conditions, among others, which makes it a very versatile and, most of all, rapidly changing sector according to the country where it is developed. These profiles cause a very different and concerning compromise on the conception of the risk faced by each of the employees involved, leading to, in many cases, low rates of occupational diseases and accidents.^3^

This is reflected in a report by the International Labor Organization, as observed by Matabanchoy-Salazar & Díaz-Bambula,^4^ who showed that, during 2014, there were approximately 6300 deaths a day, nearly 2.3 million a year, and that, according to 2019 projections, more than a half of the 321 thousand fatal accidents derive from agriculture. Additionally, the National Institute for Occupational Safety and Health mentions that^5^: “Farmers are at very risk for fatal and nonfatal injuries, work-related lung diseases, noise-induced hearing loss, skin diseases, and certain types of cancer associated with use of chemical products and prolonged sun exposure”.

Agricultural workers suffer more from pain in the back, shoulder, arms, and hands than any other health problem. Only in California more than 3 thousand agricultural workers are reported to have back injuries, with an estimated cost above $22 million dollars per year in workers’ compensation alone and it is likely that there are many more injuries that go unreported.^6^

In relation to Colombia during the first semester of 2020 there were 211,055 qualified occupational accidents, of which 13% accounted for events related specifically to the sector of agriculture, livestock, hunting, and forestry, making it the sector with the highest accident rate for the period, with 6.8 occupational accidents for every 100 workers. With regard to occupational diseases, during the first semester of 2020, out of a total of 6,074 occupational diseases, 353 were reported in the agricultural sector, accounting for 6%.^7^

These figures are very similar to those observed in South America, where it was shown that agriculture had the third highest accident rate, with an estimated rate of 10.7 accidents per 100,000 population.^8^ Furthermore, a report on occupational accidents founds that in South America the accident rate for the agricultural sector is 78.1% and that during 2019 there were 48 deaths caused by occupational accidents, and accidents and incidents resulted in absenteeism that accumulated a total of 1.2 million work days not worked.

Although figures are very expressive, there are many challenges,^9^ arising especially from:

- Little protection by the labor law, since there no parameters for this sector.
- In some countries with legislation in place, few audit process have been conducted to verify its application.
- Lack of skilled personnel to assist in the implementation of improvement actions.
- Workers’ lack of knowledge about their rights, obligations, and responsibilities.
- High rates of illiteracy, leading to more tedious processes at the time of formulating standard implementation processes, in addition to difficulties in accessing some workplaces, due to their geographical location.

Despite negative figures, it is important to face from different perspectives the development of actions aiming to formulate processes of occupational safety and health (OSH) that allow for the full development of occupational health in this sector; therefore, this investigation seeks to reveal how OSH has evolved in the scientific context from the agricultural sector perspective and what elements, factors, or processes are being conducted with the purpose of defining actions to be taken to ensure the implementation of a safe, reliable, and accessible system.

## METHODS

For the development of this research, it was defined that the databases to used would be Scopus and Web of Science, since, according to Martín-Martín et al.,^10^ they are among the most cited and referenced databases worldwide, and searches were performed using the following keywords: occupational safety and health in the agricultural sector.

Once defined the keywords, time criteria were established, limiting the search to the period from 2017 to 2022. Considering these elements, the search equation was formulated as follows: title-abs-key (occupational and health and safety and in and agriculture) and (limit-to (pubyear, 2022) or limit-to (pubyear, 2021) or limit-to (pubyear, 2020) or limit-to (pubyear, 2019) or limit-to (pubyear, 2018) or limit-to (pubyear, 2017).

With regard to inclusion and exclusion criteria, the following conditions were specified:

- Documents were filtered from 2017 to 2022.
- The title in Spanish should contain the words *seguridad y salud en el trabajo* and *sector agrícola*; in English the selected words were occupational safety and health in agriculture, with the purpose of covering as many articles as possible.
- The following keywords were established: occupational safety and health in the agricultural sector.
- Documents that were not articles were excluded, in order to define an equality criterion for the different types of documents found.
- Finally, we selected, based on their abstracts, articles that included methodologies for the implementation or follow-up of actions aimed to maintain occupational health safety in the agricultural sector (Figure 1).
  **Figure 1:**
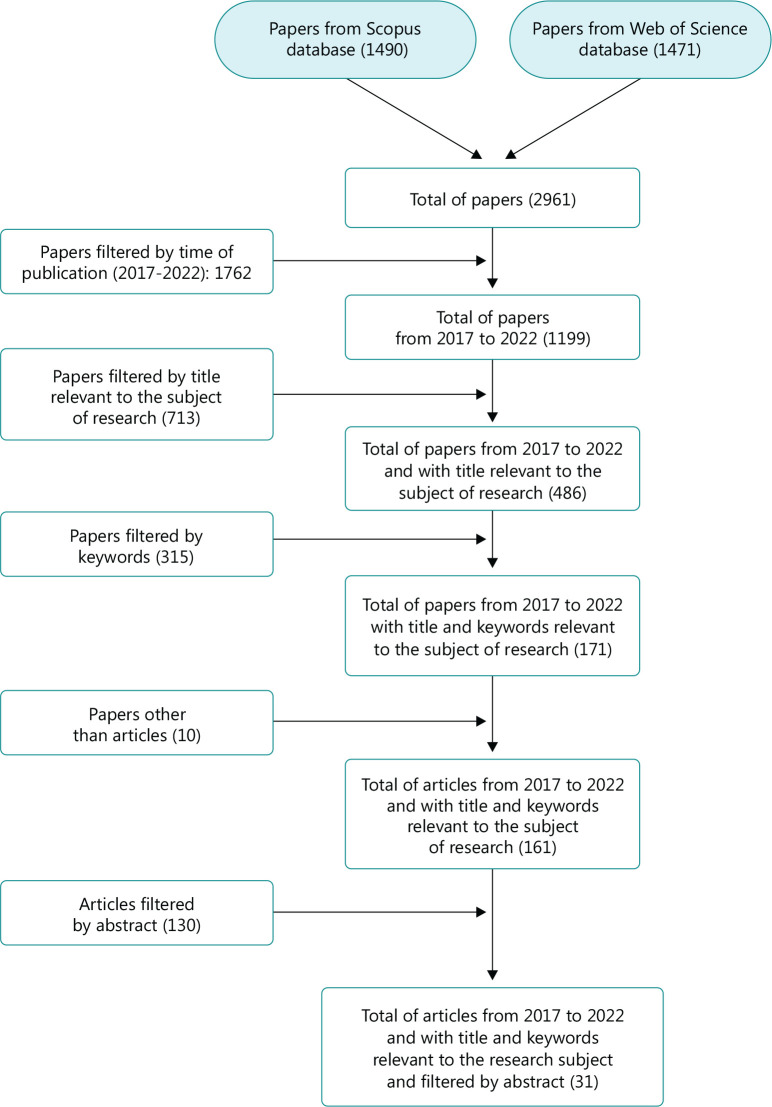
Flowchart of inclusion and exclusion criteria for article selection.

Matrices for the analysis of the final articles were integrated through author, type of crops, journal, methodology, conclusions, country, and population object of the study. Information was collected using the Biblioshiny software, from which data were exported to Excel, and filtering procedures made it possible to consolidate information, in addition to allowing for comparisons between articles to detect duplicate documents. This information was shared with specialists in bibliometric analysis and, based on their comments, the accuracy of each stage was corroborated, and the risk of bias in the selection of documents was supported and minimized. This was also validated through processes of compliance with the study objective and development of improvement alternatives according to the retrieved documents, in which positive and negative aspects were defined by the research team.

## RESULTS

### COUNTRY OF ORIGIN

In relation to country of origin, most articles regarding OSH (41%) are concentrated in the United States of America, with France in the second position, with a much lower percentage of 7%, and Italy in the third position, with 7%, followed by Canada, with 5%; South America is represented by Colombia and Chile, with a percentage of 0.3% and 0.2%, respectively.

### YEAR OF PUBLICATION

With regard to year of publication, approximately 148 documents were published in 2017, accounting for 12% of the total, while in 2021 there were 264 published documents, accounting for 22% of overall articles and representing the year with the largest amount of research published, a fact that could be associated with processes experienced due to the pandemic, which was a health issue. The years of 2020, 2019 and 2018 accounted for nearly 18% of total publications, with 203, 211 and 204 documents, respectively.

### TYPE OF CROPS

**Figure 2:**
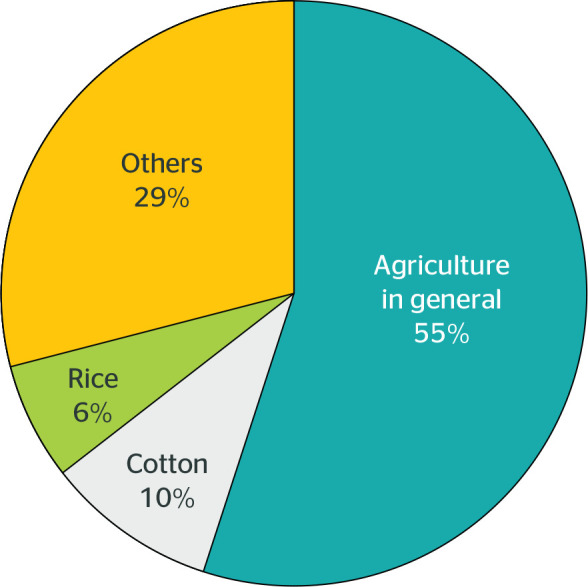
Distribution of crops found in the documents analyzed.

The type of crops observed in the analyzed articles was distributed as shown in Figure 2.

It was observed that most studies did not establish a specific crop, but rather developed the topic in function of the analysis in different areas, which enables to infer that the processes established within their results make it possible to outline a clear picture of evidence related to the sector in general terms.

### METHODOLOGY APPLIED

Various methodologies were used to collect information. Of these, the survey was the predominant technique, representing 82% of the approach used; it was defined on basis of several methods; there most important of which were direct survey, telephone survey and, finally, surveys conducted at sites related to OSH, such as clinics and hospitals.

As shown in Figure 1, 31 documents were selected, which reflect methodologies to define and establish actions that aimed to improve OSH, evidencing the following results:

- In 26% of studies, learning was identified the main driver for the development of alternatives to provide workers with knowledge and awareness on good practices that promote safe conditions; the most prominent methodologies in this area are:
  - Development of a social participation program aiming to improve knowledge on OSH through a learning process.
  - The development of a video describing OSH processes as a learning strategy; this was evaluated in comparison with other alternatives and provided evidence of its efficacy as a means of learning.
  - The university as a strategy to disseminate OSH processes through its faculty.
- Another methodology applied was social work, through the creation of focused working groups including members of the same sector, the agricultural one in this case. This alternative was found in the documents; being mentioned in 10% of them. Such methodology is focused on the development of working groups that would be created based on the personnel that would receive the intervention and that, once implemented, would allow for the application of social work techniques, in which social cards and field diaries are used to establish actions to improve OSH processes.
- Technology is also essential in the development of methodologies to improve social security; it is used in 10% of the works retrieved, with the use of a mobile application standing out within this methodology:
  - Use of OSHA-NIOSH Heat Safety Tool app.^11^
  - Use social media as a strategy to disseminate the processes to improve OSH among the agricultural community.
  - Use of print and online magazines and journal to disseminate prevention measures in OSH.
- In addition to these methodologies, there is the certified farm approach, which represents 6% of the share. The purpose of this method is to certify farms that comply with minimum OSH requirements and then provide for incentives that motivate the agricultural community to also engage in certification processes.
- Also present in 6% of the studies, there is the development of visual information as a mean to promote safe conditions, although it is worth clarifying that these were punctual conditions; for instance, this strategy was used to prevent the risk of falls due to uneven surfaces and to teach immigrants who were not fluent in the language of the country where they lived. This strategy made it possible to reduce accident rates.
- The remaining strategies were focused on different contexts; however, there were no similarities that allowed to group them together, accounted for approximately 42% of the activities developed; among them, the most important are:
  - A study conducted in Ghana with rice farmers found that religiosity has an influence of behavioral processes regarding development and compliance of OSH practices.
  - Implementation of processes and procedures to organize work for the development of better alternative to manage OSH; in other words, this methodology is focused on the development of method studies.
  - Use of checklists as a mechanism of data collection and development of alternative solutions.
  - Fear and threats as situations not to be used for the development of actions directed to formulate processes to understand and apply OSH procedures.

Table 1^12-45^ shows the articles analyzed according to title, reference number, and author.

**Table 1:** Articles analyzed according to title, reference number, and author

Author	Title	Reference
Asamani et al.	Religiosity and safety performance: mediating role of safety behaviour	(^12^)
O’Connor et al.	Safer tomorrow: Irish dairy farmers’ self-perception of their farm safety practices	(^13^)
Cook et al.	Agricultural injury surveillance using a regional trauma registry	(^14^)
Arcury et al.	The abysmal organization of work and work safety culture experienced by North Carolina Latinx women in farmworker families	(^15^)
Grzywacz et al.	Comparative effectiveness of training alternatives for the EPA’s worker protection standard regulation among immigrant Latino farmworkers	(^16^)
Caffaro et al.	Promoting farming sustainability: the effects of age, training, history of accidents and social-psychological variables on the adoption of on-farm safety behaviors	(^17^)
Schossow et al.	Building resilient agricultural communities: a process for addressing mental health challenges in agricultural communities	(^18^)
Nagami & Suenaga	Dermatitis in greenhouse farmers caused by Acaricide Cyflumetofen - an interview study	(^19^)
Du et al.	Factors associated with musculoskeletal discomfort in farmers and ranchers in the U.S. central states	(^20^)
Rohlman et al.	Evaluation of an online training for supervisors of young agricultural workers	(^21^)
Kobashi et al.	The increase in frequency of protective behavior against pesticide poisoning in Narail, Bangladesh through use of an easy paper checklist; an interventional study	(^22^)
Arphorn et al.	Working conditions and urinalysis dipstick testing among female rice farmers: a preliminary cross-sectional study	(^23^)
Sookhtanlou et al.	Farmers’ health risk and the use of personal protective equipment (PPE) during pesticide application	(^24^)
Behnami et al.	Assessment of respiratory exposure to cypermethrin among farmers and farm workers of Shiraz, Iran	(^25^)
Jakob et al.	Occupational health and safety in agriculture - a brief report on organization, legislation and support in selected European countries	(^26^)
Lari et al.	Pesticide handling practices and self-reported morbidity symptoms among farmers	(^27^)
Key & Wheat	An exploratory study of occupational safety and health with limited resource African-American farmers	(^28^)
Constantine et al.	Why don’t smallholder farmers in Kenya use more biopesticides?	(^29^)
Achard et al.	Medico-administrative data combined with agricultural practices data to retrospectively estimate pesticide use by agricultural workers	(^30^)
Devereux	Violations of farm workers’ labour rights in post-apartheid South Africa	(^31^)
Buralli et al.	Occupational exposure to pesticides and health symptoms among family farmers in Brazil	(^32^)
Jepsen et al.	Lean on your land grant: one university’s approach to address the food supply chain workforce during the COVID-19 pandemic	(^33^)
Ramos et al.	Invisible no more: the impact of COVID-19 on essential food production workers	(^34^)
Flocks	The potential impact of COVID-19 on H-2A agricultural workers	(^35^)
Rudolphi et al.	Depression, anxiety and stress among young farmers and ranchers: a pilot study	(^36^)
Quansah et al.	Respiratory and non-respiratory symptoms associated with pesticide management practices among farmers in Ghana’s most important vegetable hub	(^37^)
Taghdisi et al.	Knowledge and practices of safe use of pesticides among a group of farmers in Northern Iran	(^38^)
Marcelino et al.	Are our farm workers in danger? Genetic damage in farmers exposed to pesticides	(^39^)
Arcury et al.	Latinx child farmworkers in North Carolina: study design and participant baseline characteristics	(^40^)
Sharma et al.	Level of endosulfan among women in Talwandi Sabo block of Southern Punjab, India	(^41^)
Sanvido et al.	A quantitative risk assessment for skin sensitizing plant protection products: linking derived no-effect levels (DNELs) with agricultural exposure models	(^42^)
Kannuri & Jadhav	Generating toxic landscapes: impact on well-being of cotton farmers in Telangana, India	(^43^)
Patel et al.	Nonfatal agricultural work-related injuries: a case study from Northeast India	(^44^)
Kaprelian et al.	Integrating agricultural injury prevention with rural pediatrics: a pilot assessment	(^45^)

## DISCUSSION

**Figure 3:**
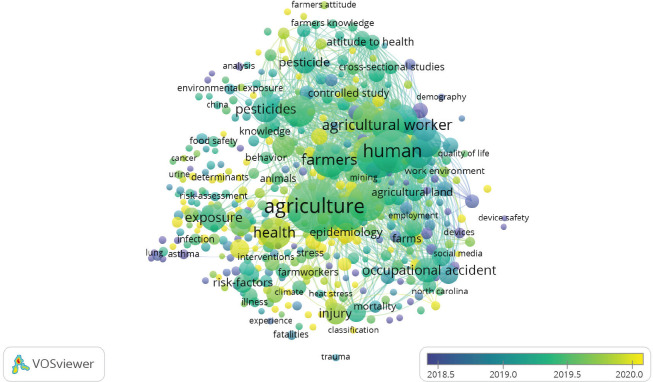
Evolution of occupational safety and health according to the keywords.

With regard to the development and evolution of OSH in the scientific context, the following graph (Figure 3) show, based on the keywords, what are the trends in the field:

In line with Figure 3, the following keywords were evidenced as trends, since they have been mostly referenced in the documents retrieved: epidemiology, safety and health, climate safety, occupational diseases, COVID-19, workplace, occupational accident, sustainability, public health, clinical trials, training, mental health, among others.

This context highlights what was established in the strategy of training as a means to generate actions to minimize risk factors within the agricultural sector. Regarding the International Labor Organization, in its recommendations say that “all workers should be provided with basic social protection. Furthermore, the workplace may be an important source of information for workers of both genders on well-being and on how to maintain a healthy lifestyle through an appropriate diet, personal hygiene, resting, and leisure”.^4^

The Instituto Nacional de Seguridad y Salud en el Trabajo,^46^ the entity that coordinates OSH in Spain within the new European Union occupational safety and health strategic framework 2021 - 2027, defines that companies should adopt digital technologies that enable to keep records of OSH conditions. These tools should also facilitate the generation of reports that contribute to more informed decision-making. In line with this, strategies that promote the use of technological platforms have been analyzed with the aim of improving OSH rates.

With regard to social participation as a strategy to improve OSH rates, the International Labor Organization^47^ promoted social dialogue as the main driver in processes to improve OSH, focusing dialogues between the government, employers, and employees, in order to general a labor culture or environment promoting compliance of standards and the formulation of policies that benefit all stakeholders.

In relation to use of technology, Lemos et al.^48^ conducted a review of trends regarding the risks associated with OSH in industry 4.0 and report, among the positive aspects, for instance, that use of technology in personal protective equipment would imply in actions that improve accident rates, since it would allow for them to have real-time data, mentioning the use of big data and the internet of things, in addition to proposing the use of machine learning in autonomous learning processes.

Within the scope of work organization, in which it could be possible to infer the relationship with studies of methods that could improve the organization of processes, Niciejewska et al.^49^ show that it is an important and determining factor in minimizing occupational accidents and incidents. One of the aspects worth highlighting is that, in terms of the differences, the production sector also assessed the possibility of causing an accident due to (inappropriate) work organization higher than the service sector.

## CONCLUSIONS

Based on the approaches established for this investigation and on the results obtained, it was possible to conclude that:

The country with the largest participation in the development of documents related to OSH in the context of the agricultural sector is the United States, which accounted for 41% of all documents found through the given search equation. With regard to year of publication, the highest number of investigations were published in 2021; finally, investigations did not focus on a specific type of crop for the development of their results, but rather focused on different crops, thus providing a clearer outline of this sector.

In relation to the methodologies developed to establish actions to improve OSH, it is worth highlighting that, in general, the survey was the most common approach to diagnose and analyze strategies, since 82% of the selected articles included this technique in their methods. Furthermore, the use of technology was found to be a means to disseminate actions to improve OSH. In addition to the aforementioned, learning is also essential to help improving OSH rates; some of the processes evidenced and considered innovative include use of religion, checklist, fear, threats, and work organization processes.

It was observed that, when asked about their health status, workers, in general, report to perform actions to maintain good health; for instance, 98% do not consume psychoactive substances, 85% report not consuming cigarettes. Therefore, heathy life habits should be encouraged and promoted, since only 51% of workers practice some type of sport.

In relation to standards, it is concerning how this normative process is being, since 75% of survey respondents, on average, reported that they are not taking any action, which is why interventions should be carried out, initiated by government entities, and supported by academic institutions to develop strategies that minimize this indicator.
